# Positivity of the English Language

**DOI:** 10.1371/journal.pone.0029484

**Published:** 2012-01-11

**Authors:** Isabel M. Kloumann, Christopher M. Danforth, Kameron Decker Harris, Catherine A. Bliss, Peter Sheridan Dodds

**Affiliations:** 1 Department of Mathematics and Statistics, University of Vermont, Burlington, Vermont, United States of America; 2 Center for Complex Systems, University of Vermont, Burlington, Vermont, United States of America; 3 Vermont Advanced Computing Center, University of Vermont, Burlington, Vermont, United States of America; 4 Department of Physics, University of Vermont, Burlington, Vermont, United States of America; Northwestern University, United States of America

## Abstract

Over the last million years, human language has emerged and evolved as a fundamental instrument of social communication and semiotic representation. People use language in part to convey emotional information, leading to the central and contingent questions: (1) What is the emotional spectrum of natural language? and (2) Are natural languages neutrally, positively, or negatively biased? Here, we report that the human-perceived positivity of over 10,000 of the most frequently used English words exhibits a clear positive bias. More deeply, we characterize and quantify distributions of word positivity for four large and distinct corpora, demonstrating that their form is broadly invariant with respect to frequency of word use.

## Introduction

While we regard ourselves as social animals, we have a history of actions running from selfless benevolence to extreme violence at all scales of society, and we remain scientifically and philosophically unsure as to what degree any individual or group is or should be cooperative and pro-social. Traditional economic theory of human behavior, for example, assumes that people are inherently and rationally selfish–a core attribute of *homo economicus*–with the emergence of global cooperation thus rendered a profound mystery [1], [2]. Yet everyday experience and many findings of psychology, behavioral economics, and neuroscience indicate people favour seemingly irrational heuristics [3], [4] over strict rationality as exemplified in loss-aversion [5], confirmation bias [6], and altruistic punishment [7]. Religions and philosophies similarly run the gamut in prescribing the right way for individuals to behave, from the universal non-harming advocated by Jainism, Gandhi's call for non-violent collective resistance, and exhortations toward altruistic behavior in all major religions, to arguments for the necessity of a Monarch [8], the strongest forms of libertarianism, and the “rational self-interest” of Ayn Rand's Objectivism [9].

In taking the view that humans are in part story-tellers–*homo narrativus*–we can look to language itself for quantifiable evidence of our social nature. How is the structure of the emotional content rendered in our stories, fact or fiction, and social interactions reflected in the collective, evolutionary construction of human language? Previous findings are mixed: suggestive evidence of a positive bias has been found in small samples of English words [10]–[12], framed as the Pollyanna Hypothesis [10] and Linguistic Positivity Bias [12], while experimental elicitation of emotional words has instead found a strong negative bias [13].

To test the overall positivity of the English language, and in contrast to previous work [11], [13], [14], we chose words based solely on frequency of use, the simplest and most impartial gauge of word importance. We focused on measuring happiness, or psychological valence [15], as it represents the dominant emotional response [16], [17]. With this approach, we examined four large-scale text corpora (see Tab. 1 for details): Twitter, The Google Books Project (English), The New York Times, and Music lyrics. These corpora, which we will refer to as TW, GB, NYT, and ML, cover a wide range of written expression including broadcast media, opinion, literature, songs, and public social interactions ([18]), and span the gamut in terms of grammatical and orthographic correctness.

**Table 1:** Details of the four corpora we examined for positivity bias.

Corpus (Abbreviation):	Date range	# Words	# Texts	Reference
Twitter (TW)	9/9/2008 to 3/3/2010	9.07 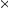 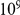	8.21 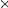 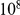 tweets	[20], [31]
Google Books Project, English (GB)	1520 to 2008	3.61 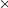 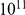	3.29 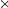 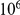 books	[32], [33]
The New York Times (NYT)	1/1/1987 to 6/30/2007	1.02 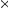 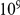	1.8 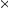 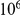 articles	[34]
Music lyrics (ML)	1960 to 2007	5.86 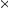 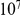	2.95 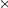 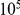 songs	[21]

We took the top 5000 most frequently used words from each corpus, and merged them to form a resultant list of 10,222 unique words. We then used Amazon's Mechanical Turk [19], [20] to obtain 50 independent evaluations per word on a 1 to 9 integer scale, asking participants to rate their happiness in response to each word in isolation (1 = least happy, 5 = neutral, and 9 = most happy [14], [21]). While still evolving, Mechanical Turk has proved over the last few years to be a reliable and fast service for carrying out large-scale social science research [22]–[26].

We computed the average happiness score and standard deviation for each word. We obtained sensible results that showed excellent statistical agreement with previous studies for smaller word sets, including a translated Spanish version (see [14], [20], [27] for details). The highest and lowest scores were 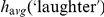 = 8.50 and 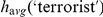 = 1.30, with expectedly neutral words averaging near 5, e.g., 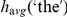 = 4.98 and 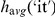 = 5.02. We refer to our ongoing studies as Language Assessment by Mechanical Turk, using the abbreviation labMT 1.0 data set for the present work (the full data set is provided as Supporting Information for [20]). Tabs. 12, 13, and 14 respectively give the top 50 words according to positivity, negativity, and standard deviation of happiness scores.

## Results and Discussion

**Figure 1:**
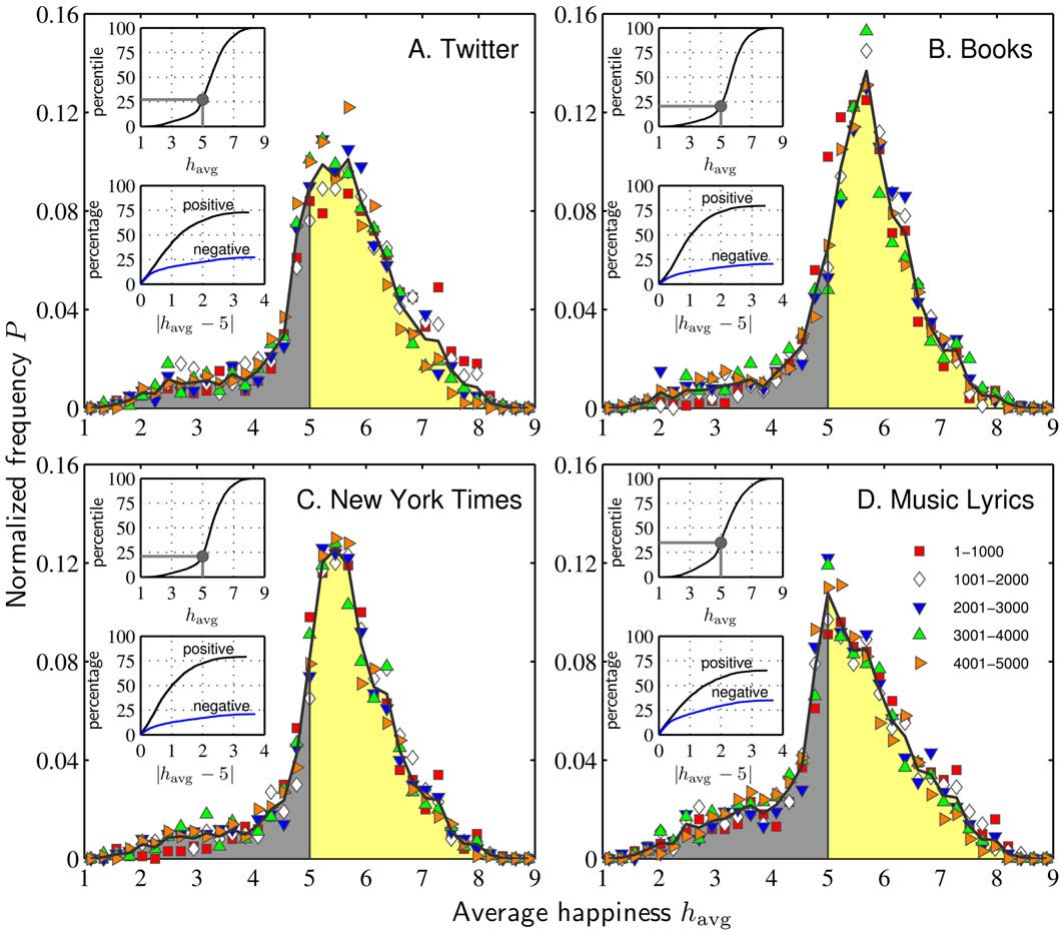
Positivity bias in the English language: normalized frequency distributions (solid black curves) of happiness scores for the 5000 most frequently used words in four corpora. Average happiness ratings for 10,222 words were obtained using Mechanical Turk with 50 evaluations per word for a total of 501,110 human evaluations (see main text). The yellow shade indicates words with average happiness scores above the neutral value of 5, gray those below. The symbols show normalized frequency distributions for words with given usage frequency ranks (see legend) suggesting a rough internal scale-free consistency of positivity Upper inset plots show percentile locations and the lower inset plots show the number of words found when cumulating toward the positive and negative sides of the neutral score of 5.

In Fig. 1, we show distributions of average word happiness 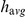 for our four corpora. We first discuss the overall distributions, i.e., those corresponding to the most frequent 5000 words combined in each corpus (black curves), and then examine the robustness of their forms with respect to frequency range. The distributions as shown were formed using 35 equal-sized bins; the number of bins does not change the visual form of the distributions appreciably, and an odd number ensures that the neutral score of 5 is a bin center. We employed binning only for visual display, using the raw data for all statistical analysis.

We see each distribution is unimodal and strongly positively skewed, with a clear abundance of positive words (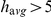, yellow shade) over negative ones (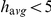, gray shade). In order, the percentages of positive words are 72.00% (TW), 78.80% (GB), 78.38% (NYT), and 64.14% (ML). Equivalently, and as further supported by Fig. 1's upper inset plots of percentile location, we see the percentile corresponding to the neutral score of 5 is well below the median. The lower inset plots show how the number of positive and negative words increase as we cumulate moving away from the neutral score of 5; positive words are always more abundant further illustrating the positive bias. The mode average happiness of words is either above neutral (TW, GB, and NYT) or located there (ML). Combining words across corpora, we also see the same overall positivity bias for parts of speech, e.g., nouns and verbs (not shown), in agreement with previous work [12].

While these overall distributions do not match in detail across corpora, we do find they have an unexpected and striking internal consistency with respect to usage frequency. We provide a series of increasingly refined and nuanced observations regarding this emotional and linguistic phenomenon of scale invariance.

First, along with the overall distribution in each plot in Fig. 1, we also show distributions for subsets of 1000 words (symbols), ordered by frequency rank 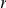 (1–1000, 1001–2000, etc.). The similarity of these distributions suggests to the eye that common and rare words are similarly distributed in their perceived degree of positivity.

In Fig. S1, we provide statistical support via 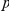-values from Kolmogorov-Smirnov tests for each pairing of distributions. Here, 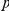-values are to be interpreted as the probability that two samples could have been derived from the same underlying distribution. The three corpora NYT, ML, and GB show the most internal agreement, and we see in all corpora that neighboring ranges of 1000 frequencies could likely match in distribution. Of the 40 pair-wise comparisons across the four corpora, 29 show statistically significant matches (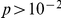).

**Figure 2:**
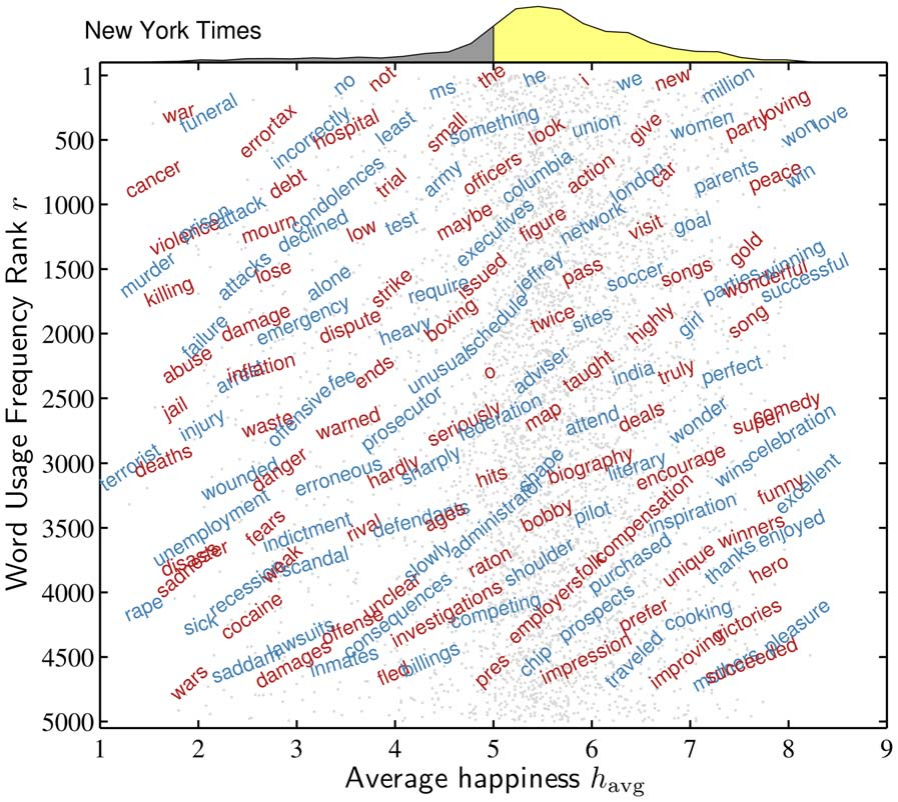
Example words for the New York Times as a function of average happiness 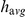 and usage frequency rank 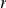. Words are centered at their values of 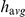 and 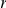, and angles and colors are only used for the purpose of readability. Each word is a representative of the set of words found in a rectangle of size 0.5 by 375 in 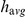 and 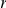, with all 5000 words located in the background by light gray points. The collapsed 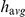 distribution at the top matches that shown in Fig. 1.

In any study of texts based on word counts, the words themselves need to be presented in some form as commonsense checks on abstracted measurements. To provide further insight into how word happiness behaves as a function of usage frequency rank, we plot a subsample of words for the New York Times in Fig. 2. We present analogous examples for the other three corpora in Figs. S2, S3, and S4. In these plots, usage frequency rank increases from bottom to top with average happiness along the bottom axis. To make clear the connection with Fig. 1, we include the overall distribution for the top 5000 words at the top of each plot. Each word is centered at the location of its values of 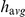 and usage frequency rank. The alternating colors are used for visual clarity only, as are the random angles. Underlying the words, the light gray points indicate the locations of all of the most frequently used 5000 words.

For the New York Times example, we find that the word pattern for average happiness and usage frequency rank is indeed reasonable. Down the right hand side of Fig. 2, we see highly positive words while decreasing in usage frequency such as ‘love’, ‘win’, ‘comedy’ ‘celebration’, and ‘pleasure’. Similarly, down the left hand side, we find ‘war’, ‘cancer’, ‘murder’, ‘terrorist’, and ‘rape’. Words of flat affect such as ‘the’, ‘something’, ‘issued’, and ‘administrator’ run down the middle of the happiness spectrum. For words with usage frequency rank near 2500, moving left to right in the plot, we find the sequence of increasingly positive words ‘jail’, ‘arrest’, ‘inflation’, ‘fee’, ‘ends’, ‘advisor’, ‘taught’, ‘india’ ‘truly’, and ‘perfect’. Moving through the space represented in other directions gives further reassurance of the general trends we observe here. Note that the random sampling of words used to generate these figures much more coarsely samples the word distributions for neutral or medium levels of happiness.

While the four corpora share common words in their most frequent 5000, numerous words appear in only one corpus. For example, ‘rainbows’ and ‘kissing’ make the top 5000 only for Music Lyrics, and ‘punishment’ the same for the Google Books corpus (see Tabs. S1 and S2). Moreover, the usage frequency rankings change strongly, as a visual comparison of Fig. 2 with Figs. S2, S3, and S4 reveals. Further detailed comparisons can be made directly from the labMT 1.0 data set [20].

To bolster our observations quantitatively, we first compute a linear regression and a Spearman correlation coefficient 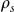 and associated 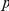-value (two-sided) for 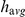 as a function of usage frequency rank, 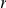. We record the results for each corpus in Tab. 2.

**Table 2:** Linear fit coefficients, Spearman correlation coefficients, and 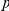-values for average word happiness 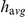 as a function of usage frequency rank 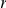.

Corpus	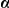	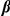	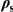	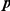 -value
Twitter	−7.78 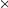 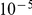	5.67	−0.103	2.3 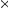 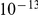
Books	−3.04 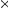 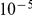	5.62	−0.013	3.5 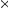 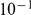
New York Times	−4.17 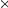 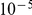	5.61	−0.0437	2.0 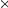 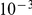
Music Lyrics:	−6.12 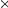 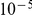	5.45	−0.0808	1.0 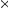 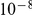

Fit is 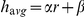.

The slopes of linear fits are all negative but extremely small, ranging from −3.04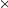 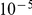 (GB) to −7.78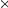 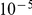 (TW). All corpora also present a weak negative correlation, ranging from 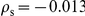 (GB) to −0.103 (TW). The correlation for the Google Books corpus is not statistically significant (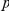 = 0.35), while it is for the other three, and especially so for TW and ML (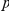 = 2.3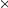 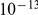 and 1.0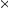 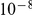).

**Figure 3:**
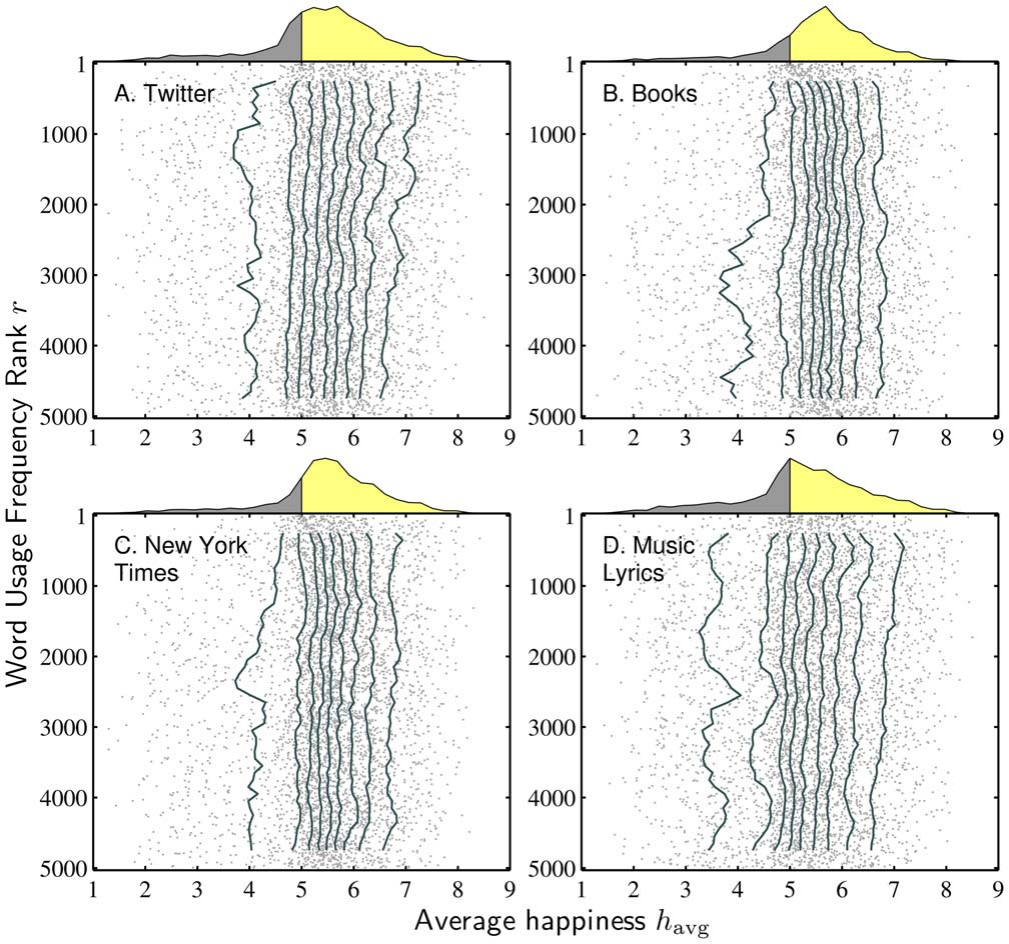
Deciles for average word happiness 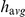 distributions as a function of word usage frequency rank 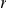. These ‘jellyfish plots’ are created using a sliding window of 500 words moving down the vertical axis of usage frequency rank in increments of 100. The gray points mark 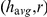 for individual words, as in Fig. 2. The overall distributions of 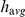, matching those in Fig. 1, cap each plot.

We next move to a more detailed quantitative view of the word happiness distribution as a function of word usage frequency. In Fig. 3, we show how deciles behave as a function of usage frequency rank. Using a sliding window containing 500 words, we compute deciles moving down the usage frequency rank axis. Using these ‘jellyfish plots’, we see that apart from the lowest decile (which is universally uneven), GB and NYT are very stable while a slight negative trend is perceptible for TW and ML. We can now with some confidence state that the measured, edited writing of the New York Times and the Google Books corpus possess a remarkable scale invariance in emotion with respect to word usage frequency. The emotional content of words on Twitter and in music lyrics, while still roughly similar across usage frequency ranks, show a small bias towards common words being disproportionately positive in comparison with increasing rare ones. The bias is sufficiently small as to be likely indiscernible by an individual familiar with these corpora; moreover, cognitive biases regarding the salience of information would presumably render such detection impossible [28].

We have thus far considered distributions of average happiness values for words. Each word's estimate comes from a distribution of assessment scores, and a useful, simple investigation can be carried out on the standard deviation of individual word happiness, 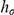.

A range of word and concept categories yielded high 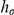 in our study, the top 50 of which are shown in Tab. S3. At the top of the list, we observe words that are or relate to profanities, alcohol and tobacco, religion, both capitalism and socialism, sex, marriage, fast foods, climate, and cultural phenomena such as the Beatles, the iPhone, and zombies. As a result of variation in the rater's preferences perhaps due to inherent controversy or cultural and demographic variation, these terms all elicited diverse responses.

**Figure 4:**
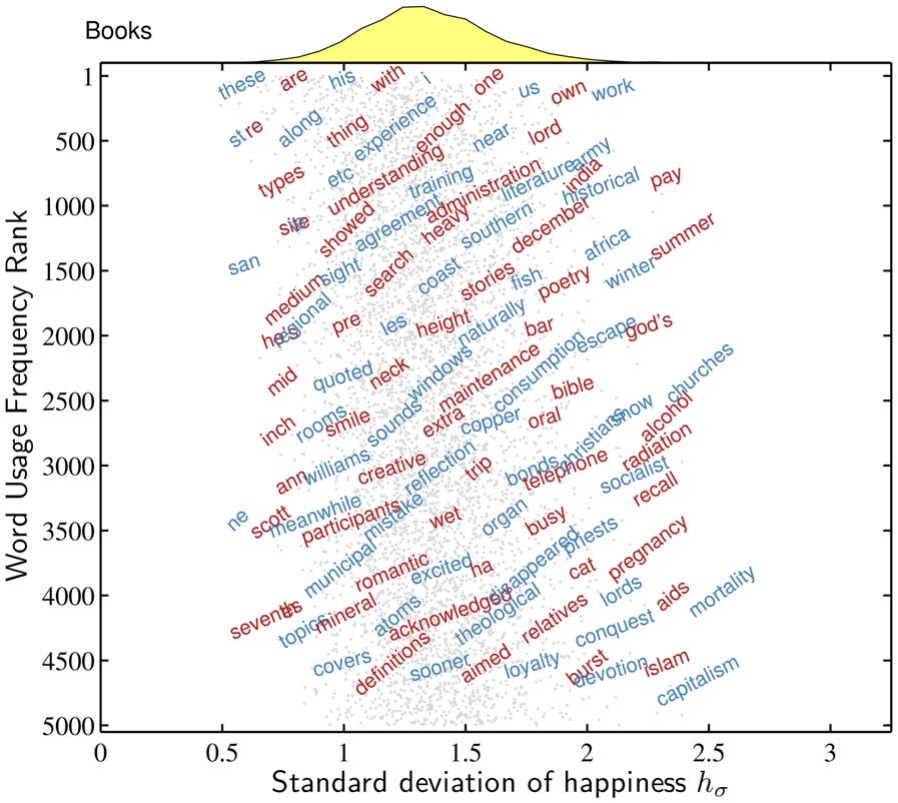
Example words for the Google Books corpus as a function of usage frequency rank and standard deviation of happiness estimates. Similar to Fig. 2, each word shown represents all words in rectangles of size 0.2 and 375 in 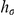 and 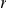. The histogram at the top of the figure represents the overall distribution for 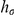 for the first 5000 most frequent words. The light gray points indicate locations of the most frequent 5000 words in the Google Books corpus.

**Figure 5:**
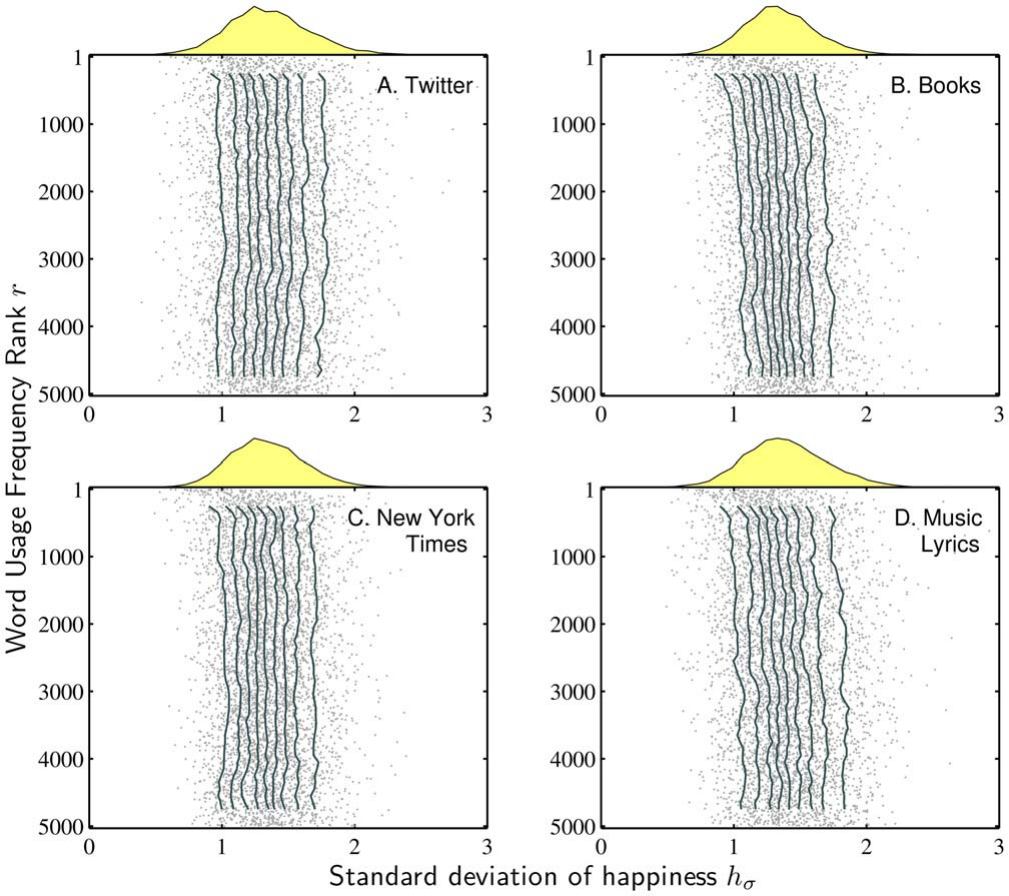
Deciles for standard deviations. As for Fig. 3, these ‘jellyfish plots’ are created using a sliding window of 500 words moving across the horizontal axis of usage frequency rank increments of 100.

We repeat our analyses of 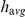 for 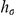 by first considering a sample of words for the Google Books corpus, Fig. 4, and then the behavior of deciles, Fig. 5. (In Fig. S5 we present the overall distributions, the equivalent of Fig. 1.) For our entire collection of words, we find most values of 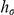 fall in the range 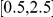.

In Fig. 4, we show example words from the Google Books corpus as a function of word usage frequency rank and standard deviation (Figs. S6, S7, and S8 show the same for TW, NYT, and ML). The right hand side of Fig. 4 shows example words with high 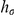 and increasing usage frequency rank including ‘work’, ‘pay’, ‘summer’, ‘churches’, ‘mortality’ and ‘capitalism’. For low 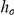 (the left hand side of Fig. 4), we see basic, neutral words such as ‘these’, ‘types’, ‘inch’, and ‘seventh’.

While this word diagram is primarily intended for qualitative purposes, we see that for 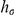, the overall trend for Google Books is a gradual increase as a function of usage frequency rank. In other words, relatively rarer words have higher standard deviations in comparison with relatively more common ones. This is confirmed visually in Fig. 5, where we present jellyfish plots showing deciles for all four corpora. The Music Lyrics corpus shows a similar increase in 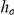 with usage frequency rank as GB, whereas TW and NYT corpora exhibit no obvious linear variation. These observations are supported by the linear fits and Spearman correlation coefficients recorded in Tab. 3, where we consider 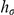 as a function of usage frequency rank. All linear approximations yield a very small positive growth, with both the TW and NYT corpora clearly smaller than the other two, particularly TW. The corresponding Spearman correlation coefficients indicate we have statistically significant monotonic growth in 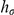 for GB, ML, and NYT, particularly the first two, and indicates no evidence of growth for TW.

**Table 3:** Spearman correlation coefficients for standard deviation of word happiness estimates as a function of usage frequency rank.

Corpus	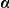	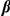	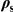	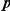 -value
Twitter	1.47 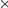 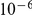	1.35	0.0116	4.1 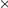 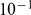
Books	3.36 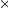 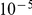	1.27	0.176	5.0 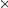 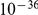
New York Times	9.33 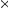 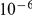	1.32	0.0439	1.9 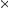 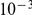
Music Lyrics	2.76 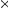 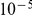	1.33	0.134	1.6 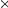 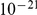

Fit is 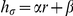.

All told, we find slight deviation from an exact scaling independence of 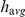 and 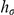 in terms of usage frequency rank, but it is highly constrained and corpus specific. In particular, the corpora that show a slight negative correlation between 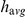 and usage frequency rank, TW and ML, do not match those showing a positive correlation between 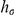 and usage frequency rank, GB and ML.

Our findings are that positive words strongly outnumber negative words overall, and that there is a very limited, corpus-specific tendency for high frequency words to be more positive than low frequency words. These two aspects of positivity and usage frequency can only be separated with the kind of data we study here. Previous claims that positive words are used more frequently [10]–[12], suffered from insufficient, non-representative data. For example, Rozin et al. recently compared usage frequencies for just seven adjective pairs of positive-negative opposites [11]. Augustine et al. showed that average happiness and usage frequencies for 1034 words [14] were more positively correlated than we observe here [12]; however, since these words were chosen for their meaningful nature [14], [29], [30] rather than by their rate of occurrence, their findings are naturally tempered. A positivity bias is also not inconsistent with many observations that negative emotions in isolation are more potent and diverse than positive words [28].

In sum, our findings for these diverse English language corpora suggest that a positivity bias is universal, that the emotional spectrum of language is very close to self-similar with respect to frequency, and that in our stories and writings we tend toward prosocial communication. Our work calls for similar studies of other languages and dialects, examinations of corpora factoring in popularity (e.g., of books or articles), as well as investigations of other more specific emotional dimensions. Related work would explore changes in positivity bias over time, and correlations with quantifiable aspects of societal organization and function such as wealth, cultural norms, and political structures. Analyses of the emotional content of phrases and sentences in large-scale texts would also be a natural next, more complicated stage of research. Promisingly, we have shown elsewhere for Twitter that the average happiness of individual words correlates well with that of surrounding words in status updates [20].

## Supporting Information

Figure S1
**Results of Kolmogorov-Smirnov tests comparing word happiness distributions shown in** **Fig. 1** **.** For each corpus, the 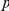-value reports the probability that the two samples being compared could come from the same distribution with lighter colors meaning more likely. The gray-scale corresponds to 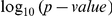.(TIFF)

Figure S2 **Example words for Twitter as a function of usage frequency rank and average happiness.**
(TIFF)

Figure S3 **Example words for the Google Books corpus as a function of usage frequency rank and average happiness.**
(TIFF)

Figure S4 **Example words for the Music Lyrics corpus as a function of usage frequency rank and average happiness.**
(TIFF)

Figure S5 **Overall distributions of standard deviations in happiness scores for the four corpora.** As with average happiness, distributions for subsets of usage frequency ranks (symbols, see legend).
(TIFF)

Figure S6 **Example words for Twitter as a function of usage frequency rank and standard deviation of happiness estimates.**
(TIFF)

Figure S7 **Example words for the New York Times as a function of usage frequency rank and standard deviation of happiness estimates.**
(TIFF)

Figure S8 **Example words for the Music Lyrics corpus as a function of usage frequency rank and standard deviation of happiness estimates.**
(TIFF)

Table S1 **The 50 most positive words, as assessed by our Mechanical Turk survey.** Rankings of each word in the four corpora are provided. A ‘–’ indicates a word was not in the most frequent 5000 words in the given corpus.
(PDF)

Table S2 **The 50 most negative words in our data set.**
(PDF)

Table S3 **The top 50 words according to the standard deviation of happiness estimates.**
(PDF)
