# Supplementary figures and images for: Positivity of the English Language
Source: PLoS One. 2012 Jan 11;7(1):e29484. doi: 10.1371/journal.pone.0029484 (PMC3256157; doi:10.1371/journal.pone.0029484)

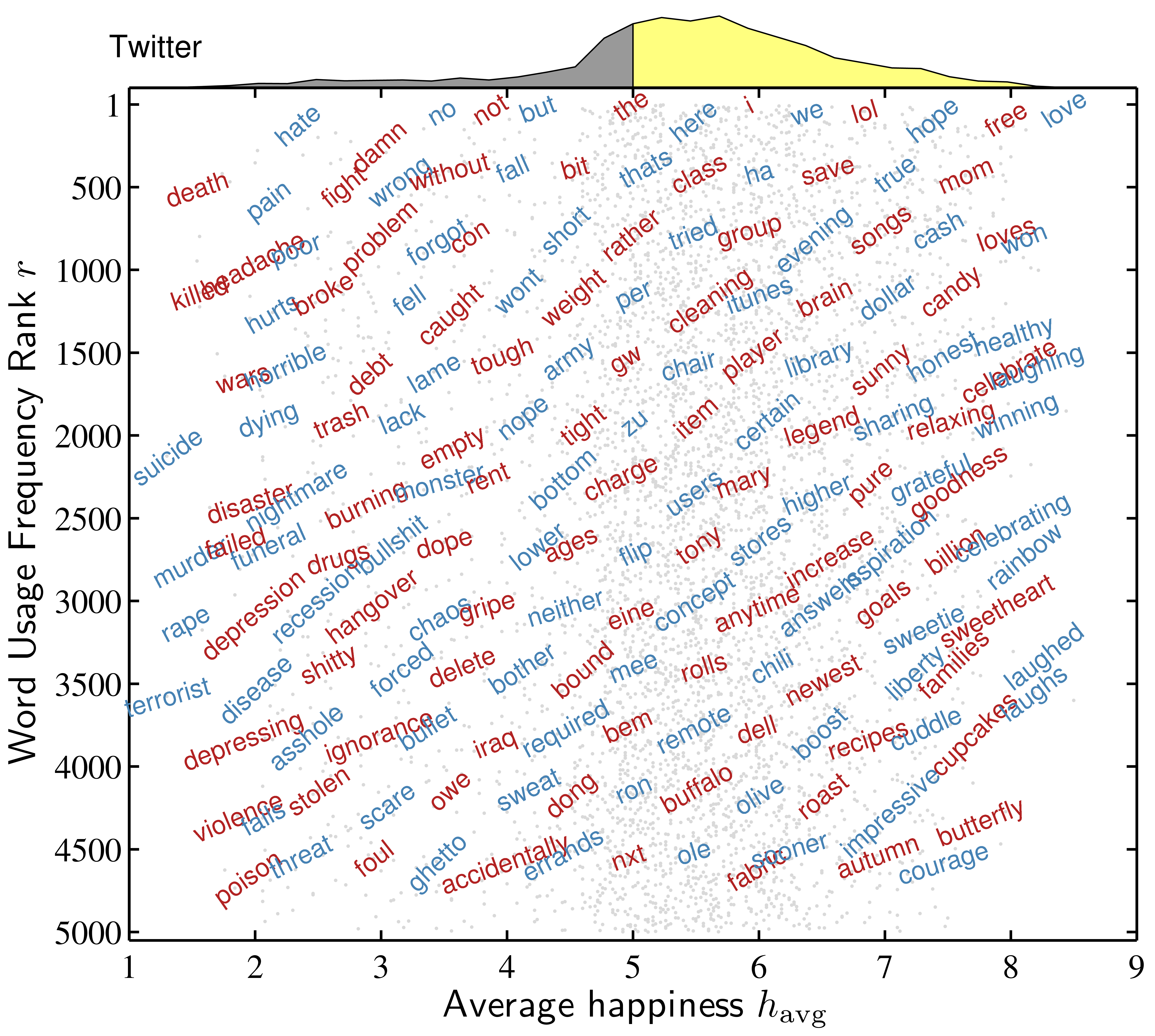

Supplement: Figure S2 — Example words for Twitter as a function of usage frequency rank and average happiness. (TIFF) [file pone.0029484.s002.tiff]

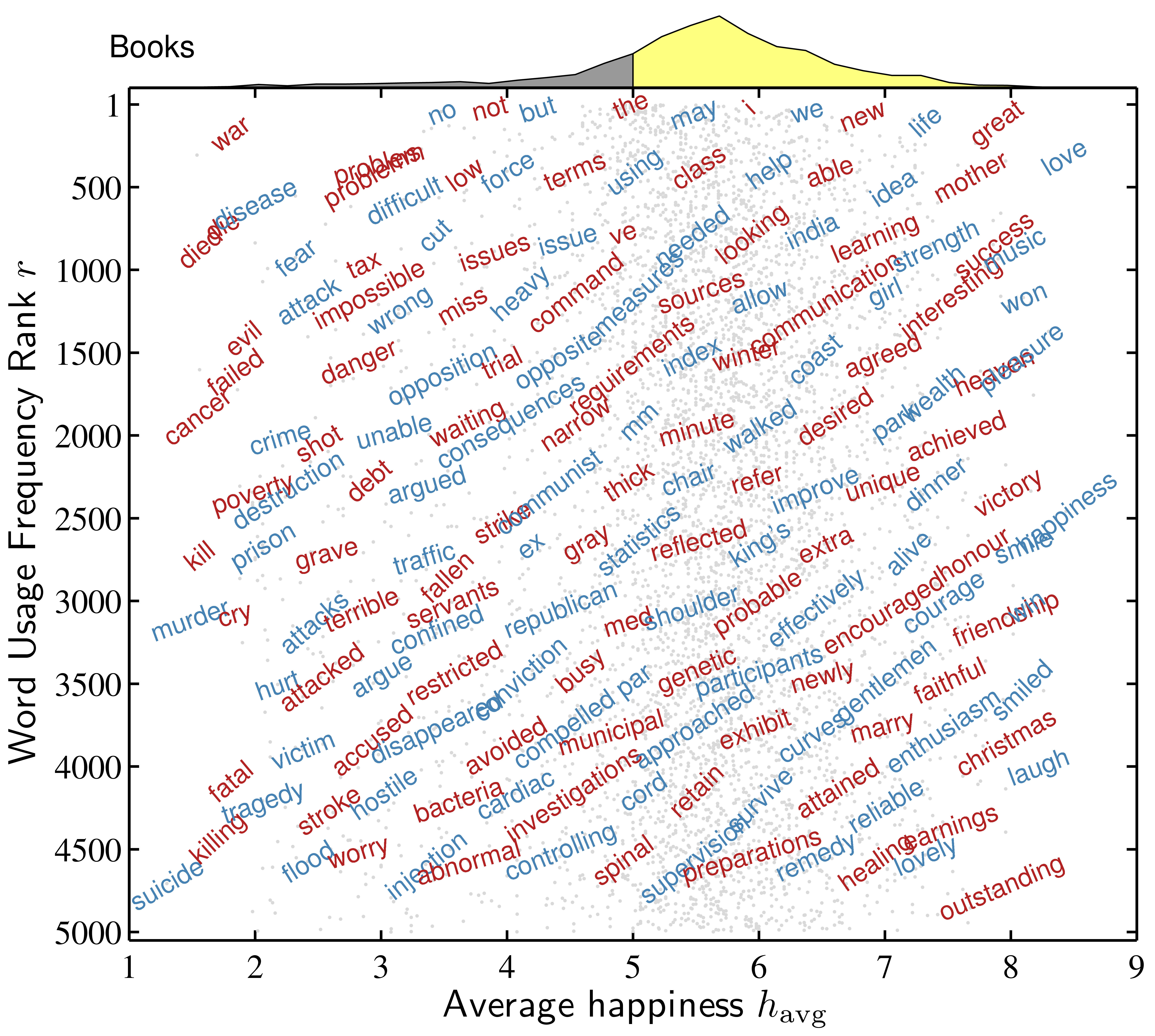

Supplement: Figure S3 — Example words for the Google Books corpus as a function of usage frequency rank and average happiness. (TIFF) [file pone.0029484.s003.tiff]

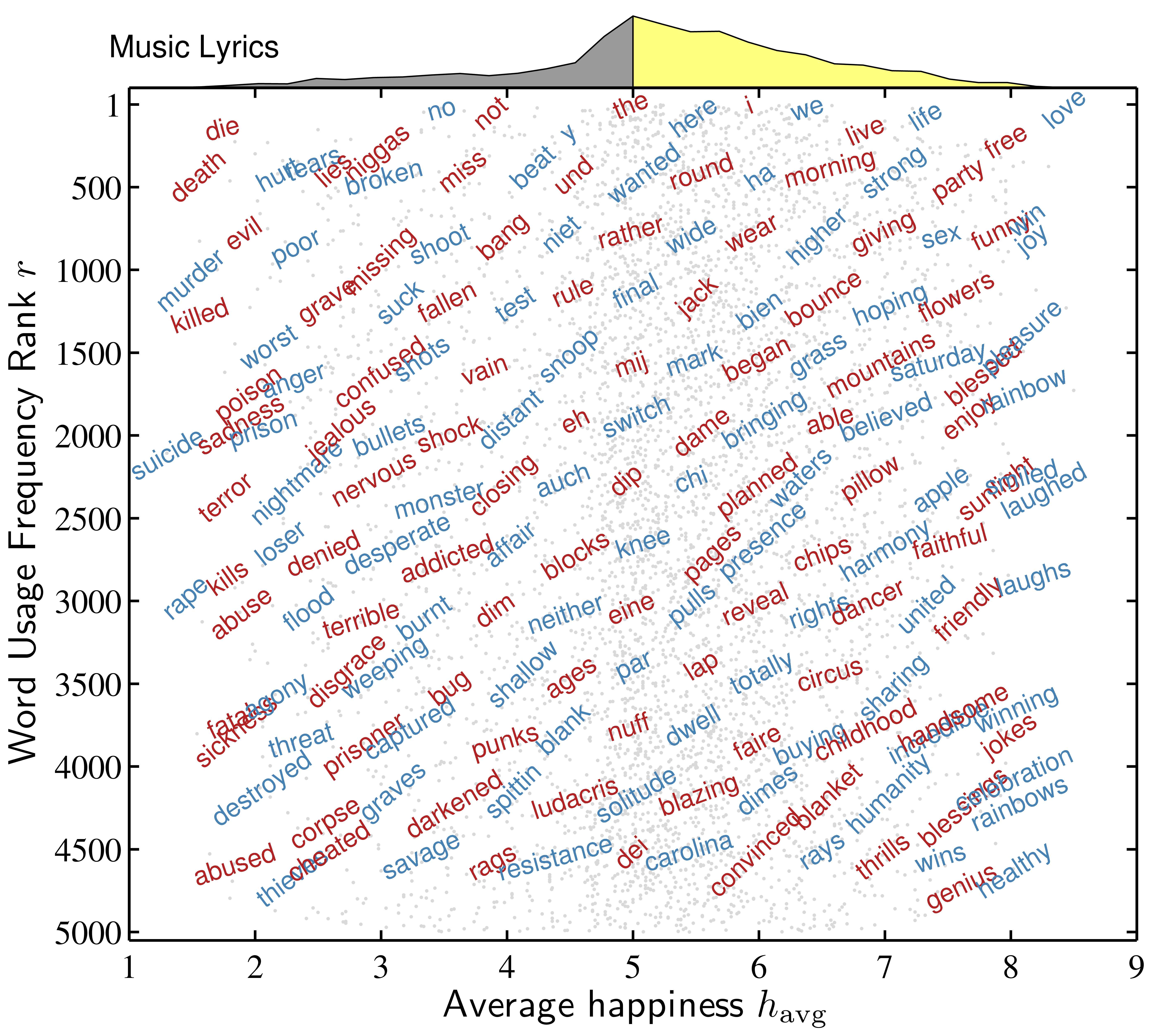

Supplement: Figure S4 — Example words for the Music Lyrics corpus as a function of usage frequency rank and average happiness. (TIFF) [file pone.0029484.s004.tiff]

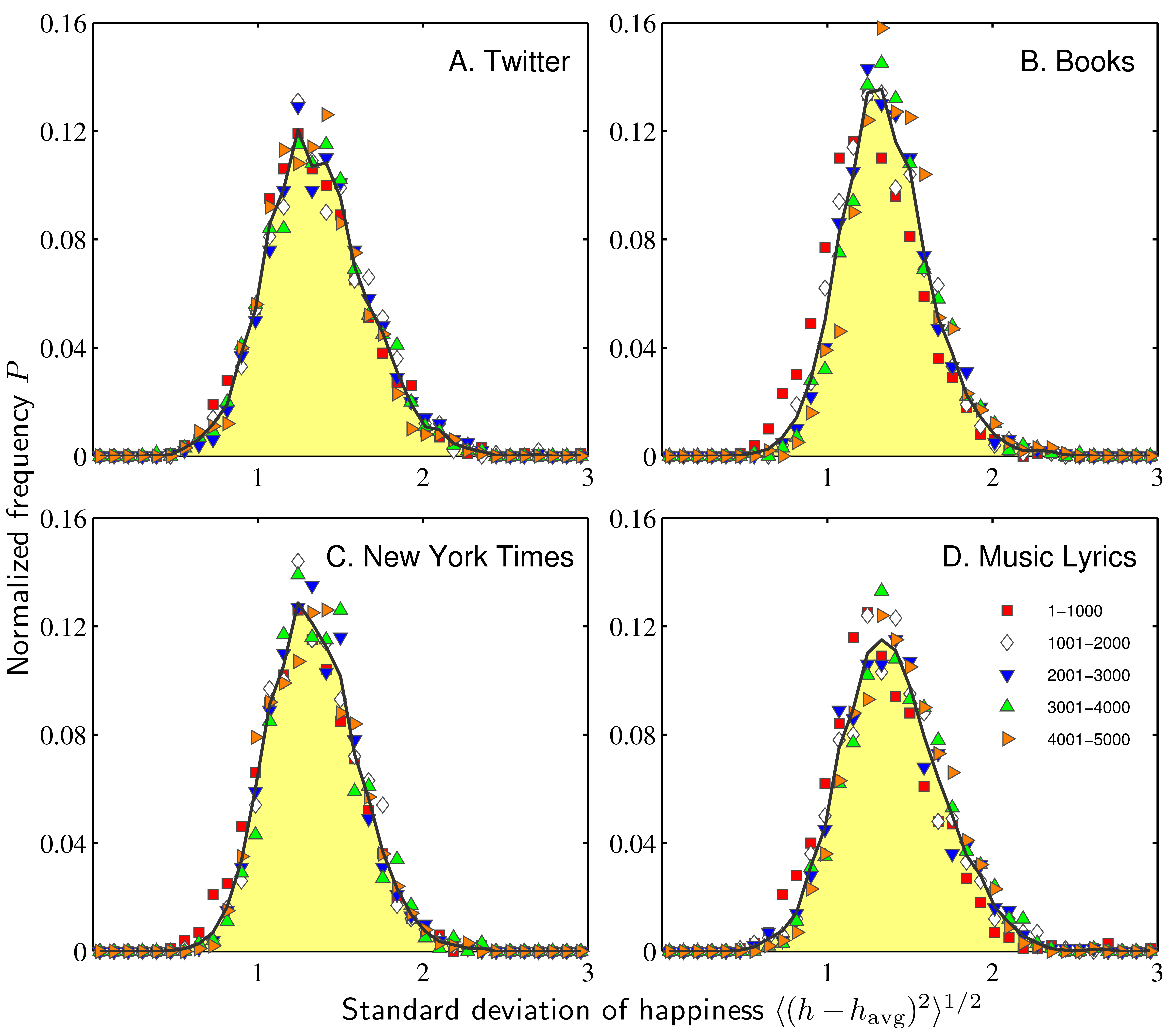

Supplement: Figure S5 — Overall distributions of standard deviations in happiness scores for the four corpora. As with average happiness, distributions for subsets of usage frequency ranks (symbols, see legend). (TIFF) [file pone.0029484.s005.tiff]

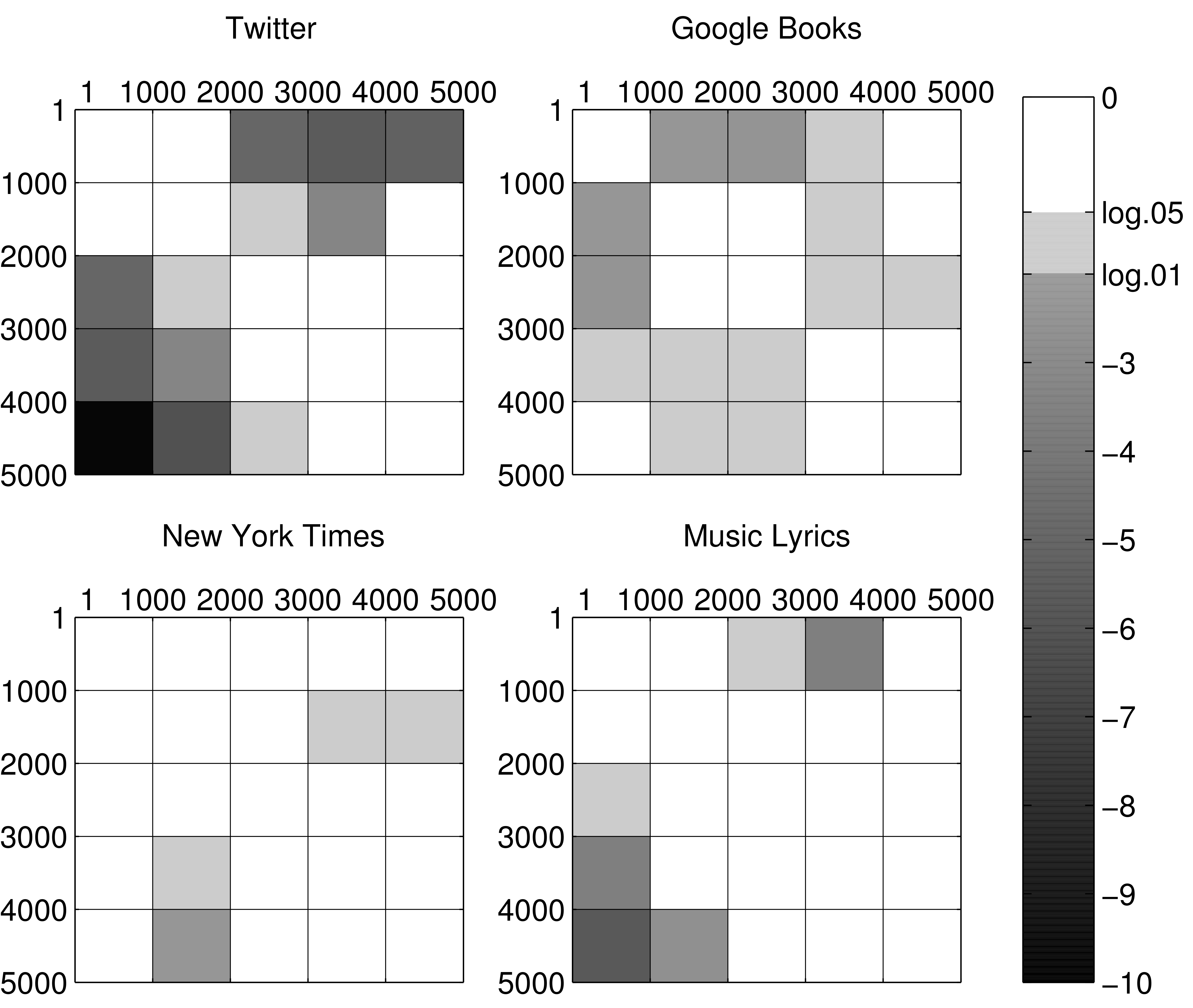

Supplement: Figure S6 — Example words for Twitter as a function of usage frequency rank and standard deviation of happiness estimates. (TIFF) [file pone.0029484.s006.tiff]

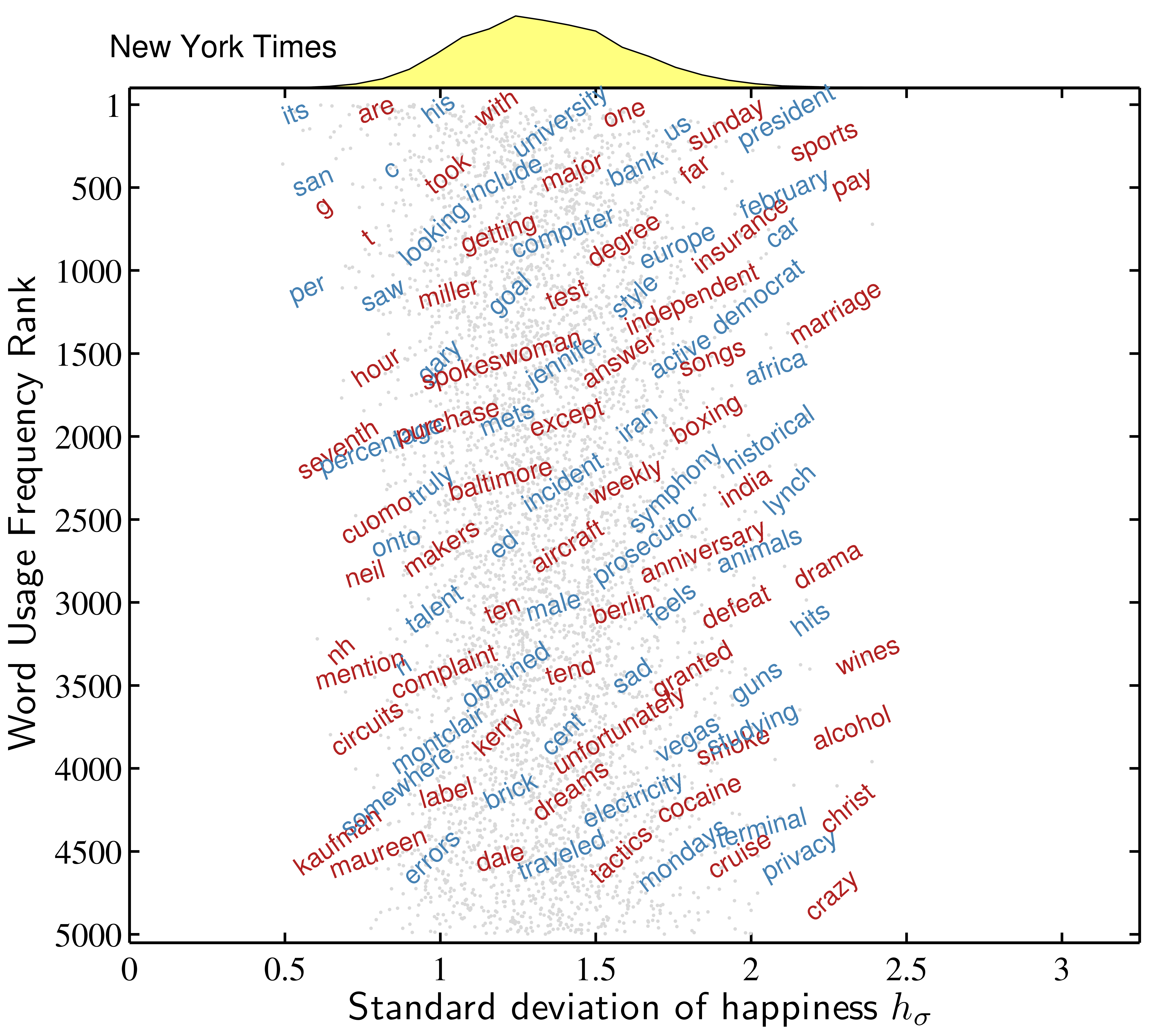

Supplement: Figure S7 — Example words for the New York Times as a function of usage frequency rank and standard deviation of happiness estimates. (TIFF) [file pone.0029484.s007.tiff]

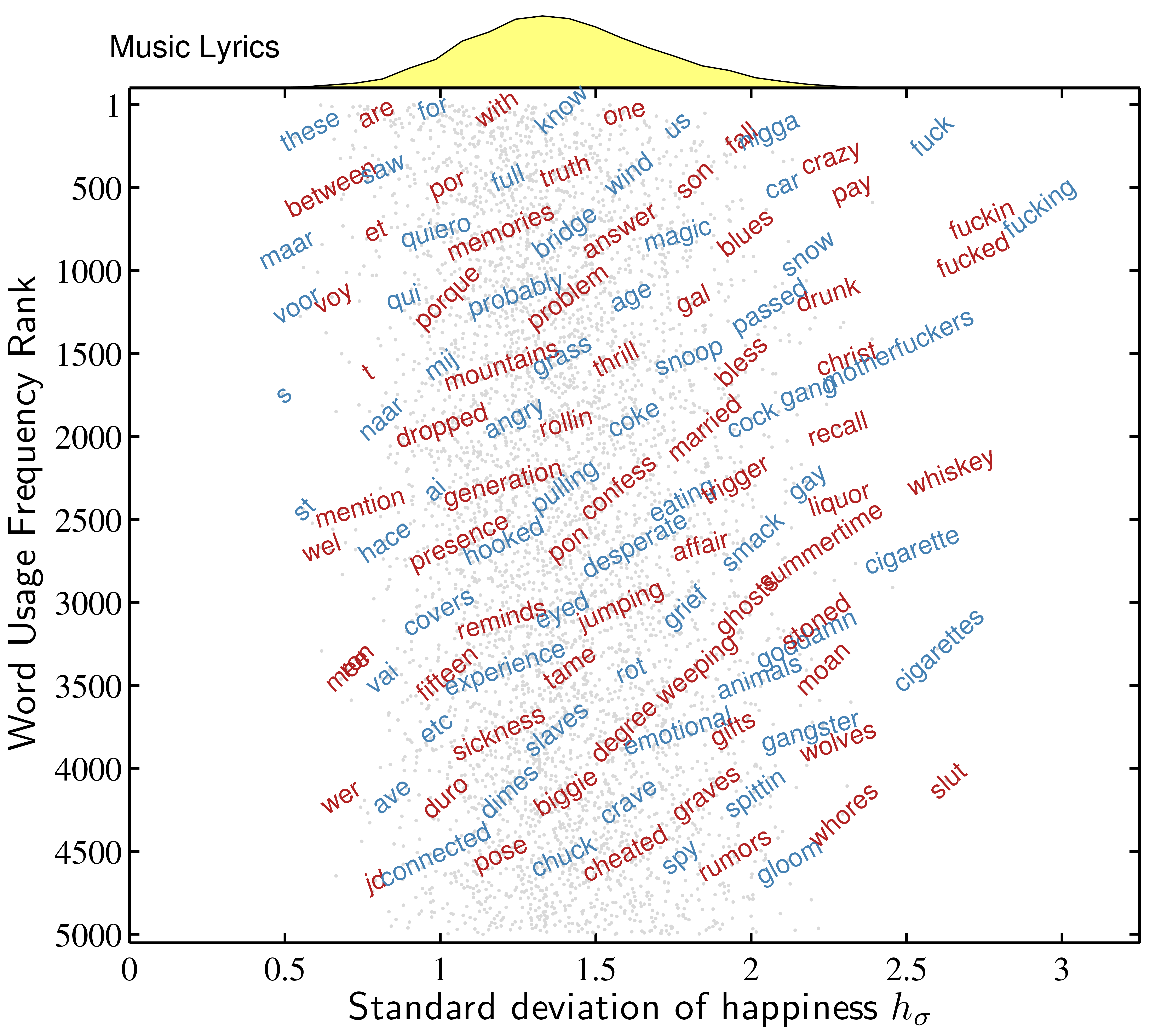

Supplement: Figure S8 — Example words for the Music Lyrics corpus as a function of usage frequency rank and standard deviation of happiness estimates. (TIFF) [file pone.0029484.s008.tiff]
