# Supplementary material for: Positivity of the English Language
Source: PLoS One. 2012 Jan 11;7(1):e29484. doi: 10.1371/journal.pone.0029484 (PMC3256157; doi:10.1371/journal.pone.0029484)
Supplement: Table S1 — The 50 most positive words, as assessed by our Mechanical Turk survey. Rankings of each word in the four corpora are provided. A ‘–’ indicates a word was not in the most frequent 5000 words in the given corpus. (PDF) [file pone.0029484.s009.pdf]

h_{rank}	word	h_{avg}	h_{σ}	TW rank	GB rank	NYT rank	ML rank
1	laughter	8.50	0.9313	3600	–	–	1728
2	happiness	8.44	0.9723	1853	2458	–	1230
3	love	8.42	1.1082	25	317	328	23
4	happy	8.30	0.9949	65	1372	1313	375
5	laughed	8.26	1.1572	3334	3542	–	2332
6	laugh	8.22	1.3746	1002	3998	4488	647
7	laughing	8.20	1.1066	1579	–	–	1122
8	excellent	8.18	1.1008	1496	1756	3155	–
9	laughs	8.18	1.1551	3554	–	–	2856
10	joy	8.16	1.0568	988	2336	2723	809
11	successful	8.16	1.0759	2176	1198	1565	–
12	win	8.12	1.0812	154	3031	776	694
13	rainbow	8.10	0.9949	2726	–	–	1723
14	smile	8.10	1.0152	925	2666	2898	349
15	won	8.10	1.2164	810	1167	439	1493
16	pleasure	8.08	0.9655	1497	1526	4253	1398
17	smiled	8.08	1.0660	–	3537	–	2248
18	rainbows	8.06	1.3603	–	–	–	4216
19	winning	8.04	1.0490	1876	–	1426	3646
20	celebration	8.02	1.5318	3306	–	2762	4070
21	enjoyed	8.02	1.5318	1530	2908	3502	–
22	healthy	8.02	1.0593	1393	3200	3292	4619
23	music	8.02	1.1156	132	875	167	374
24	celebrating	8.00	1.1429	2550	–	–	–
25	congratulations	8.00	1.6288	2246	–	–	–
26	weekend	8.00	1.2936	317	–	833	2256
27	celebrate	7.98	1.1516	1606	–	3574	2108
28	comedy	7.98	1.1516	1444	–	2566	–
29	jokes	7.98	0.9792	2812	–	–	3808
30	rich	7.98	1.3169	1625	1221	1469	890
31	victory	7.98	1.0784	1809	2341	687	2845
32	christmas	7.96	1.2930	138	3846	2097	599
33	free	7.96	1.2610	85	342	393	219
34	friendship	7.96	1.1241	4273	3098	3669	3980
35	fun	7.96	1.3087	110	4135	2189	463
36	holidays	7.96	1.2610	1204	–	–	–
37	loved	7.96	1.1599	465	2178	890	517
38	loves	7.96	1.3696	780	–	–	653
39	loving	7.96	1.0093	947	4396	230	527
40	beach	7.94	1.0577	573	3596	551	1475
41	hahaha	7.94	1.5572	428	–	–	–
42	kissing	7.94	1.1323	–	–	–	2052
43	sunshine	7.94	1.1678	2080	–	–	950
44	beautiful	7.92	1.1753	266	1159	1754	467
45	delicious	7.92	1.2591	1565	–	–	–
46	friends	7.92	1.1925	258	658	347	321
47	funny	7.92	1.0467	358	–	3194	755
48	outstanding	7.92	1.1400	4468	4721	1797	–
49	paradise	7.92	1.3974	3096	–	–	1146
50	sweetest	7.92	1.2911	–	–	–	2232

Table S1. The 50 most positive words, as assessed by our Mechanical Turk survey. Rankings of each word in the four corpora are provided. A '–' indicates a word was not in the most frequent 5000 words in the given corpus.
