# Supplementary material for: Positivity of the English Language
Source: PLoS One. 2012 Jan 11;7(1):e29484. doi: 10.1371/journal.pone.0029484 (PMC3256157; doi:10.1371/journal.pone.0029484)
Supplement: Table S2 — The 50 most negative words in our data set. (PDF) [file pone.0029484.s010.pdf]

h_{rank}	word	h_{avg}	h_{σ}	TW rank	GB rank	NYT rank	ML rank
10173	disease	2.00	1.3093	3531	598	1391	1780
10174	illness	2.00	1.1780	–	2738	1690	–
10175	killers	2.00	1.5253	–	–	–	3303
10176	punishment	2.00	1.3401	–	2750	–	–
10177	criminal	1.98	1.2696	2722	2421	1322	3261
10178	depression	1.98	1.5583	3082	2406	–	–
10179	headache	1.98	1.1156	959	–	–	–
10180	poverty	1.98	1.1156	–	2343	3744	–
10181	tumors	1.98	1.3461	–	4876	–	–
10182	bomb	1.96	1.2771	1292	–	2815	1227
10183	disaster	1.96	1.4280	2399	–	3729	3355
10184	fail	1.96	1.0294	1160	2481	4030	1758
10185	poison	1.94	1.1502	4668	–	–	1740
10186	depressing	1.90	1.2164	3838	–	–	–
10187	earthquake	1.90	1.1995	2733	–	–	–
10188	evil	1.90	1.2817	975	1416	–	781
10189	wars	1.90	1.3286	1654	3252	4696	2888
10190	abuse	1.88	1.2395	2809	2865	2236	3069
10191	diseases	1.88	0.9398	–	2307	4795	–
10192	sadness	1.88	1.1891	–	–	3820	1930
10193	violence	1.86	1.0500	4299	1724	1238	2016
10194	cruel	1.84	1.1493	2963	–	–	1447
10195	cry	1.84	1.2835	1028	3075	–	226
10196	failed	1.84	0.9971	2645	1618	1276	2920
10197	sickness	1.84	1.1843	4735	–	–	3782
10198	abused	1.83	1.3101	–	–	–	4589
10199	tortured	1.82	1.4241	–	–	–	4693
10200	fatal	1.80	1.5253	–	4089	–	3724
10201	killings	1.80	1.5386	–	–	4914	–
10202	murdered	1.80	1.6288	–	–	–	4796
10203	war	1.80	1.4142	468	175	291	462
10204	kills	1.78	1.2337	2459	–	–	2857
10205	jail	1.76	1.0214	1642	–	2573	1619
10206	terror	1.76	1.0012	4625	4117	4048	2370
10207	die	1.74	1.1920	418	730	2605	143
10208	killing	1.70	1.3590	1507	4428	1672	998
10209	arrested	1.64	1.0053	2435	4474	1435	–
10210	deaths	1.64	1.1386	–	–	2974	–
10211	raped	1.64	1.4251	–	–	–	4528
10212	torture	1.58	1.0515	3175	–	–	3126
10213	died	1.56	1.1980	1223	866	208	826
10214	kill	1.56	1.0529	798	2727	2572	430
10215	killed	1.56	1.2316	1137	1603	814	1273
10216	cancer	1.54	1.0730	946	1884	796	3802
10217	death	1.54	1.2811	509	307	373	433
10218	murder	1.48	1.0150	2762	3110	1541	1059
10219	terrorism	1.48	0.9089	–	–	3192	–
10220	rape	1.44	0.7866	3133	–	4115	2977
10221	suicide	1.30	0.8391	2124	4707	3319	2107
10222	terrorist	1.30	0.9091	3576	–	3026	–

Table S2. The 50 most negative words in our data set.
