# Supplementary material for: Positivity of the English Language
Source: PLoS One. 2012 Jan 11;7(1):e29484. doi: 10.1371/journal.pone.0029484 (PMC3256157; doi:10.1371/journal.pone.0029484)
Supplement: Table S3 — The top 50 words according to the standard deviation of happiness estimates. (PDF) [file pone.0029484.s011.pdf]

h_{rank}	word	h_{avg}	h_{σ}	TW rank	GB rank	NYT rank	ML rank
8426	fucking	4.64	2.9260	448	–	–	620
9263	fuckin	3.86	2.7405	1077	–	–	688
9469	fucked	3.56	2.7117	1840	–	–	904
8020	pussy	4.80	2.6650	2019	–	–	949
3770	whiskey	5.72	2.6422	–	–	–	2208
9462	slut	3.57	2.6300	–	–	–	4071
9652	cigarettes	3.31	2.5997	–	–	–	3279
9043	fuck	4.14	2.5794	322	–	–	185
8797	mortality	4.38	2.5546	–	3960	–	–
9767	cigarette	3.09	2.5163	–	–	–	2678
10050	motherfuckers	2.51	2.4675	–	–	–	1466
3801	churches	5.70	2.4599	–	2281	–	–
9985	motherfucking	2.64	2.4558	–	–	–	2910
6390	capitalism	5.16	2.4524	–	4648	–	–
9015	porn	4.18	2.4302	1801	–	–	–
1516	summer	6.40	2.3905	896	1226	721	590
2914	beer	5.92	2.3891	839	4924	3960	1413
9759	execution	3.10	2.3889	–	2975	–	–
1830	wines	6.28	2.3737	–	–	3316	–
9179	zombies	4.00	2.3733	4708	–	–	–
8898	aids	4.28	2.3477	2983	3996	1197	–
7839	capitalist	4.84	2.3418	–	4694	–	–
9370	revenge	3.71	2.3363	–	–	–	2766
2716	mcdonalds	5.98	2.3342	3831	–	–	–
1400	beatles	6.44	2.3313	3797	–	–	–
8348	islam	4.68	2.3250	–	4514	–	–
5785	pay	5.30	2.3234	627	769	460	499
6205	alcohol	5.20	2.3212	2787	2617	3752	3600
9818	muthafuckin	3.00	2.3094	–	–	–	4107
2145	christ	6.16	2.3067	2509	909	4238	1526
10016	motherfuckin	2.58	2.3043	–	–	–	1562
2074	burger	6.18	2.3008	2070	–	–	–
6931	thunder	5.06	2.2983	3681	–	–	1313
9592	whores	3.40	2.2946	–	–	–	4275
3016	naked	5.90	2.2879	1317	4908	–	1343
4347	#iphone	5.58	2.2865	–	–	–	–
5481	liquor	5.36	2.2836	4915	–	–	2372
9553	radiation	3.45	2.2827	–	2847	–	–
8416	wolves	4.65	2.2781	–	–	–	3835
8511	recall	4.60	2.2768	4770	3177	4105	1950
5625	walmart	5.34	2.2733	2817	–	–	–
7414	socialism	4.96	2.2727	–	4605	–	–
961	marriage	6.70	2.2700	2444	1050	1246	–
9882	bombs	2.86	2.2679	–	–	–	2867
2920	christianity	5.92	2.2663	–	2554	–	–
4549	vodka	5.56	2.2602	3606	–	–	–
8420	crazy	4.64	2.2566	383	–	4761	312
5345	sushi	5.40	2.2497	2232	–	–	–
3385	god's	5.80	2.2497	–	1915	–	–
9251	drunk	3.88	2.2464	1006	–	–	1140

Table S3. The top 50 words according to the standard deviation of happiness estimates.
